# Carbon Nanotube–Polyurethane Composite Sheets for Flexible Thermoelectric Materials

**DOI:** 10.1021/acsanm.3c03247

**Published:** 2023-09-19

**Authors:** Antonio J. Paleo, Yadienka Martinez-Rubi, Beate Krause, Petra Pötschke, Michael B. Jakubinek, Behnam Ashrafi, Christopher Kingston

**Affiliations:** † 2C2T-Centre for Textile Science and Technology, University of Minho, 4800-058 Guimarães, Portugal; ‡ Security and Disruptive Technologies Research Centre, National Research Council Canada, Ottawa, Ontario K1A 0R6, Canada; § Leibniz-Institut für Polymerforschung Dresden e.V. (IPF), Hohe Str. 6, 01069 Dresden, Germany; ∥ Aerospace Research Centre, National Research Council Canada, 5145 Decelles Avenue, Montreal, Quebec H3T 2B2, Canada

**Keywords:** carbon nanotubes, thermoplastic polyurethane, nanocomposite fabrics, thermal conductivity, thermoelectric properties

## Abstract

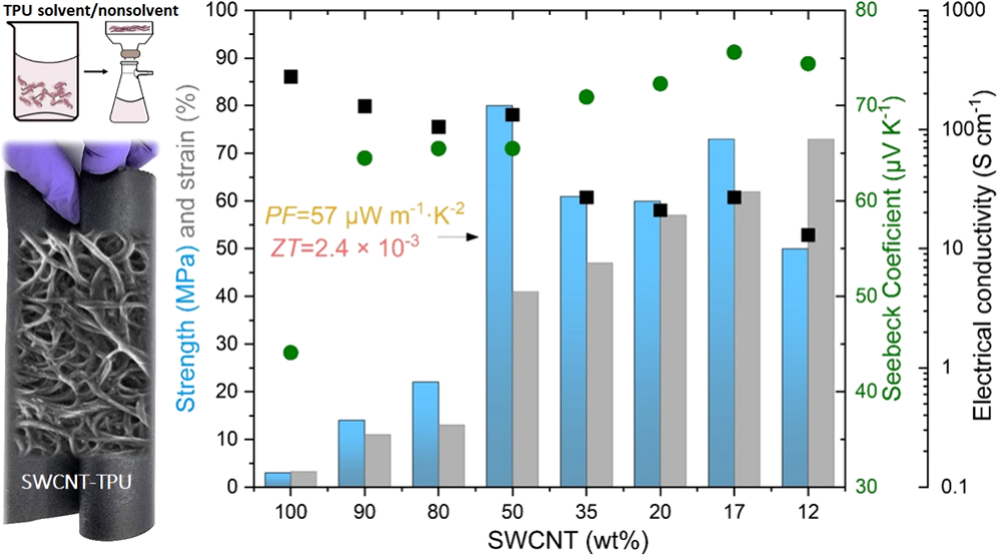

Integration of single-wall carbon nanotubes (SWCNTs) in the form of fabriclike sheets or other preformed assemblies (films, fibers, etc.) simplifies their handling and allows for composites with higher nanotube contents, which is needed to better exploit their outstanding properties and achieve multifunctional materials with improved performance. Here, we show the development of p-type SWCNT–thermoplastic polyurethane (TPU) fabric materials with a wide range of SWCNT contents (from 5 to 90 wt %) by employing a one-step filtration method using a suspension of SWCNTs in a TPU solvent/nonsolvent mixture. The mechanical and thermoelectric (TE) properties of these SWCNT–TPU nanocomposites were tailored by varying the SWCNT/TPU wt % ratio, achieving significant advantages relative to the pristine SWCNT buckypaper (BP) sheets in terms of strength and stretchability. In particular, the SWCNT–TPU nanocomposite with a 50/50 wt % ratio composition (equivalent to 15 vol % of SWCNTs) shows a power factor (PF) of 57 μW m^–1^ K^–2^, slightly higher compared to the PF of the SWCNT BP prepared under the same conditions (54 μW m^–1^ K^–2^), while its mechanical properties significantly increased (e.g., ∼7-, 25-, and 250-fold improvements in stiffness, strength, and tensile toughness, respectively). These results represent a significant step toward the development of easy-to-process self-supporting and stretchable materials with robust mechanical properties for flexible thermoelectric devices.

## 1 Introduction

Thermoelectric (TE) materials are able to transform a temperature difference into a movement of charge carriers to induce electrical current and voltage or, alternatively, use the flow of electricity to provide for solid-state cooling. The former offers a promising approach to generate useful energy from low-grade heat sources, in particular waste heat.^1^ The efficiency of a TE material is determined by the dimensionless figure of merit (*ZT*), , with σ as the electrical conductivity, *k* as the thermal conductivity, *T* as the absolute temperature, and *S* as the Seebeck coefficient calculated by , with Δ*V* representing the voltage obtained at determined temperature gradient Δ*T*.^2^ The sign of *S* (negative or positive) depends on the majority charge carrier. If *S* is positive, the TE material (p-type) is characterized by an electrical conduction dominated by holes, whereas for an n-type TE material, the *S* is negative and the majority charge carriers are electrons.^3^ The challenge of a significant thermoelectric effect is the simultaneous combination of high *S*, high σ, and low *k*. In this respect, inorganic semiconductors such as Bi_2_Te_3_ and their derivatives, which yield *ZT* > 1 near room temperature, have been intensively studied.^4^ However, because of their scarcity, difficult processability, mechanical stiffness, and high cost, the interest in seeking different types of TE materials has increased over the last decades.^5^

Conductive polymer composites (CPCs), such as those consisting of nonconductive polymers and carbon nanotubes (CNTs), are becoming subject of research due to the compromise provided by the relatively low k and high flexibility of the polymer component as well as the excellent power factor (PF = *S*^2^ σ) of CNTs.^6^ Notably, a literature survey in this topic reveals that most TE materials based on polymers consist of intrinsically conducting polymers (ICPs), namely, poly(3,4-ethylenedioxythiophene)-poly(styrenesulfonate) (PEDOT:PSS),^7−9^ poly(3-hexylthiophene) (P3HT),^10^ and others. This is due to the high electrical conductivity and excellent biocompatibility of ICPs, even if their mechanical properties and stretchability are limited.^11^ In contrast, although the mechanical properties of polyurethane (PU) should represent an ideal choice for flexible applications because of its high stretchability along with good tensile strength, impact performance, and corrosion and abrasion resistance,^12^ there are few studies focused on the TE and mechanical properties of PU/CNT composites. In this regard, Xiao et al. described the preparation of CPCs based on single-wall carbon nanotubes (SWCNTs) wrapped on polymer particles synthesized by emulsion polymerization and a water-dispersible PU with blocked terminal isocyanate groups (PUBI) as a flexible component and cross-linking agent.^13^ Their polymer composites with 10 wt % of SWCNTs achieved σ = 110 S cm^–1^, *S* = 24 μV K^–1^, and PF = 0.52 μW m^–1^ K^–2^, with an elongation at break of 3.8%. In another work, Tzounis et al. reported three-dimensional (3D) printed melt-mixed thermoplastic polyurethane (TPU) composites with different wt % of multiwall carbon nanotubes (MWCNTs). The composites with 5 wt % MWCNTs exhibited σ = 0.94 S cm^–1^, *S* = 18.5 μV K^–1^, PF = 0.04 μW m^–1^ K^–2^, and *ZT* = 1.42 × 10^–5^, with Young’s modulus (*E*) of 28.5 MPa, tensile strength of 10.4 MPa, and elongation at break of 161%.^14^ Notably, both works describe the production of CPCs with low to moderate contents of CNTs. This is because high CNT loadings often pose processing challenges or lead to poor CNT dispersion and low mechanical properties. On the other hand, high content of CNTs is useful to better leverage their functional properties, including to increase the electrical conductivity that is advantageous for TE materials.

SWCNT buckypapers (BPs), which consist of nominally 100 wt % SWCNTs and are obtained by filtration of SWCNT dispersions, have also been applied as TE materials. For example, Hewitt et al. evaluated the TE properties of SWCNT BP and solution-mixed composite thin films of poly(vinylidene fluoride) (PVDF) with a wide SWCNT concentration range (5–75 wt %), achieving a figure of merit in the range from 7 × 10^–6^ for the SWCNT BP to 10^–4^ for the PVDF film with 5 wt % of SWCNTs; however, their mechanical properties were not evaluated.^15^ When applying such buckypapers, typically both the p-type and the n-type are enhanced by various low-molecular-weight additives.^16−20^ In this direction, Nonoguchi et al.^17^ have conducted a screening experiment with different additives, which includes phosphine and imine-containing molecules as dopants, and the *S* of pristine SWCNT films increased from 49 to 90 μV K^–1^ by addition of carbazole, while the PF of p-type films raised from 8 μW m^–1^·K^–2^ for pristine SWCNT films up to 25 μW m^–1^·K^–2^, and a *ZT* of 0.078, when doped with tetracyanoquinodimethane (TCNQ). In another work, polymeric dopants were utilized for SWCNT BP modification by immersing them overnight in various solutions.^18^ Thus, an *S* of 50 μV K^–1^ was achieved after immersing the SWCNT BP in a polystyrene–chloroform solution. Moreover, Hata et al. studied surfactant-wrapped nanotubes to generate an n-type TE material,^21^ which achieves a PF = 240 μW m^–1^·K^–2^ and a *ZT* of 6 × 10^–3^.

Mytafides et al. demonstrated a novel ink-dispensing/printing process to fabricate an all-carbon thermoelectric-printed generator based on SWCNT Tuball films.^22^ The p-type films were stabilized by sodium dodecylbenzenesulfonate (SDBS), and the n-type films were obtained by addition of cetyltrimethylammonium bromide (CTAB) and printed using the blade-coating technique on a Kapton substrate. Remarkable PFs of 145 and 127 μW m^–1^·K^–2^ at room temperature for the p- and n-type films, respectively, were achieved. By applying such inks and using a mask-assisted specified circuit architecture, a TEG with 116 p-/n pairs and a power output of 345 μW was generated.

However, despite all of these significant high values of *S* and PF, all of the above-modified SWCNT materials, as BPs, infiltrated or modified BPs, or printed inks, have safety concerns associated with direct exposure of the CNTs on the surfaces of the materials or TEGs and cannot compete with TE materials based on CNT–polymer composites in terms of mechanical properties. In particular, for flexible thermoelectric generators^23^ and wearable thermoelectric generators (WTGEs),^22^ the need for materials that offer not only high *ZT* and flexibility but also stretchability has been recognized.^24,14^ Using the human body as a heat source, due to the arbitrary geometry of the surface and static and moving objects, requires high degrees of mechanical deformability (>10%) for WTEGs. However, strain values of only <1% for inorganic metals and <5% for conductive polymers can be achieved.^25^ The described ink-printed TEG^22^ based on SWCNTs is flexible, but not stretchable due to the stiff substrate.

For the applications of CPCs in flexible and stretchable TEG applications, it is crucial to analyze the effects of the SWCNT concentration to obtain the best possible combination between TE and mechanical properties. In the present work, both mechanical and TE properties are analyzed in SWCNT BP and SWCNT–TPU composite sheets with a wide range of SWCNT content (5 to 90 wt %). The obtained self-supporting sheets were fabricated by adapting a recently reported one-step filtration method developed for the fabrication of high-nanotube-content nanocomposites using MWCNTs and a TPU adhesive.^26^

Specifically, the method uses a TPU solvent/nonsolvent combination to enhance polymer interaction with nanotubes and to facilitate a fast recovery of nonwoven composite sheets of controlled composition by vacuum filtration.^26,27^ With this method, the adsorption of TPU on SWCNTs can be controlled through solubility modulation, and TPU chains form a stable shell or coating around dispersed SWCNT/SWCNT bundles. The recovered SWCNT–TPU composites described in this study are nonwoven fabriclike sheets made of highly entangled and randomly oriented TPU/SWCNT “nanofibers”. This method of producing nanocomposite sheets is in contrast to more common processing methods that involve first the fabrication of a high-nanotube-content preform or buckypaper, followed by a polymer infiltration step, as well as methods that involve solution casting, followed by slow solvent evaporation to produce films.^3^ In this way, the morphology, mechanical properties, and TE properties of CPCs with a broad range of SWCNT contents produced by this innovative method are presented.

## 2 Experimental Section

### 2.1 Materials

Single-wall carbon nanotubes (SWCNTs) under the brand name Tuball (OCSiAl, Luxembourg) were purchased and used as received. The selection was based on a former study comparing different kinds of CNTs.^28^ These SWCNTs have diameters of 1–3 nm and lengths of 1–5 μm.^29^ According to the manufacturer, the SWCNT powder contains less than 15% of metal impurities. The thermoplastic polyurethane (TPU) was an ester-based polyurethane (Pine Brook, NJ) with a density of 1.19 g cm^–3^ and a Shore hardness of 85A.

### 2.2 Preparation of SWCNT BP and Nonwoven SWCNT–TPU Composite Sheets

**Figure 1:**
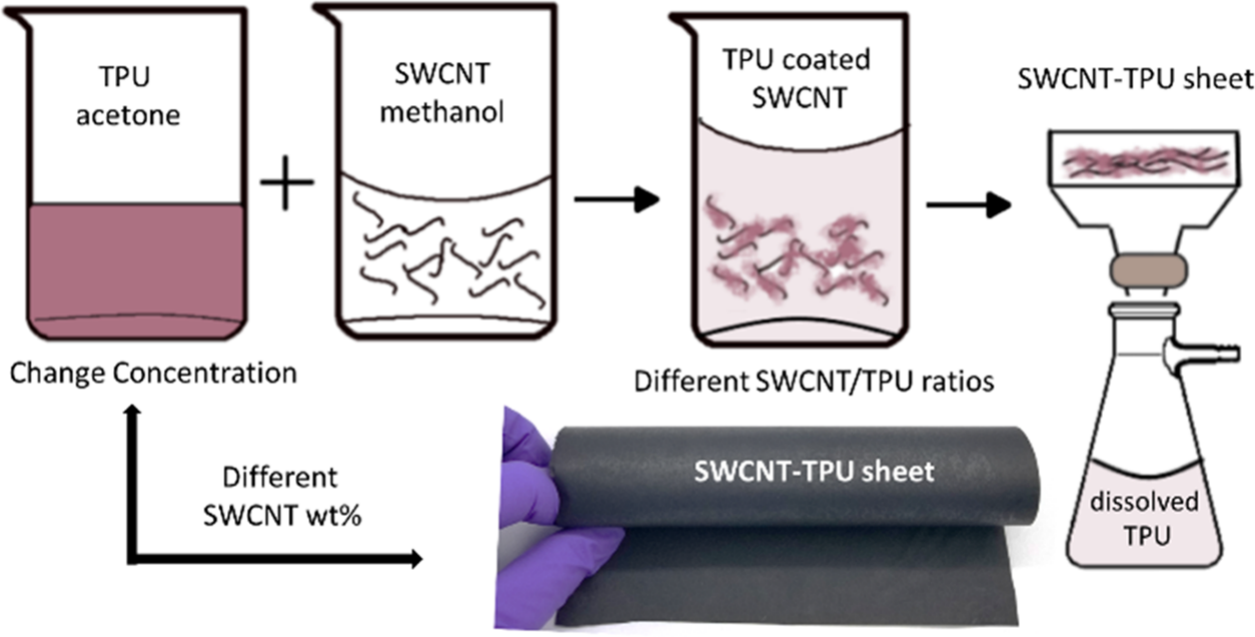
Schematic of the one-step filtration method to fabricate nonwoven SWCNT–TPU composite sheets. SWCNT contents between 5 and 90 wt % are achieved by increasing the TPU concentration in acetone (i.e., changing the SWCNT/TPU ratio in the acetone/methanol mixture) while keeping SWCNT mass constant.

Nonwoven SWCNT–TPU composite sheets with high loadings of SWCNTs were fabricated by a previously reported one-step filtration method.^26^ The fabrication method is illustrated in Figure 1. Briefly, about 600 mg of SWCNTs was suspended in 1 L of methanol using an IKA 25 Ultra-Turrax high-speed disperser for 5 min, bath sonication (Branson 8800) for 1 h followed by horn sonication (Misonix, 15% output, 30% duty cycle) for 30 min, and additional bath sonication for 1 h. The SWCNT suspensions were then added to TPU solutions of different concentrations in acetone (1 L), which contained the required amount of dissolved TPU to achieve different SWCNT/TPU weight ratios (i.e., 1:0.1 to 1:20, see Table 1). Combining the SWCNT suspension and TPU solution was facilitated by bath sonication for 1 h. During this step, the suspension was also mixed 3 times with the high-speed disperser for 2 min. Horn sonication was then applied for 30 min, followed by bath sonication for 30 min.

**Table 1:** Basic Characteristics of the SWCNT BP and Nonwoven SWCNT–TPU Composite Sheets

		recovered SWCNT–TPU nanocomposite sheets					
					volume fraction (vol %)		
sample name	SWCNT/TPU wt. ratio in solvent	SWCNT/TPU (wt % ratio)	thickness (μm)	density (g cm^–3^)	SWCNT	TPU	void
SWCNT BP	1:0	100:0	78	0.31	17	0	83
SWCNT-TPU-90	1:0.1	90:10	88	0.32	16	3	81
SWCNT-TPU-80	1:0.25	80:20	92	0.35	15	7	78
SWCNT-TPU-50	1:1.5	50:50	100	0.55	15	24	62
SWCNT-TPU-35	1:2.5	35:65	137	0.51	10	28	62
SWCNT-TPU-20	1:5	20:80	190	0.61	7	40	52
SWCNT-TPU-17	1:7	17:83	145	1.02	10	71	19
SWCNT-TPU-12	1:10	12:88	243	0.94	6	69	24
SWCNT-TPU-8	1:15	8:92	432	0.91	4	71	25
SWCNT-TPU-5	1:20	5:95	590	0.92	3	73	24

The “TPU-coated” SWCNTs were then recovered as nonwoven sheets by vacuum filtration through a PTFE membrane (10 μm pore size) using a Venturi air pump and 15 cm × 15 cm filter. The filtration was completed quickly (within 2–5 min) for all compositions evaluated. The wet nanocomposite sheets were immediately sandwiched between PTFE membranes and filter papers and dried under compression (10 MPa) in a press overnight at room temperature, after which the sheets were peeled from the filter membrane, placed between Teflon films, and further dried at 75 °C in vacuum for 10 h to remove residual solvent. More details on how the compositions presented in Table 1 were obtained are shown in the Supporting Information (SI).

### 2.3 Characterization

Scanning electron microscopy (SEM) images of the surfaces were taken with a Hitachi High Technologies S-4800v. Raman spectra were obtained at a minimum of five random locations on each sample using a Renishaw inVia Reflex Raman microscope with 514.5 nm laser excitation and a laser power of 0.3 mW at the sample and collected through a 50 × 0.75NA objective. Tensile testing was performed using an Instron 5900R loadframe with a 500 N load cell and a displacement rate of 5 mm min^–1^. A minimum of five strips (∼30 mm × 2 mm) of each material were tested. For the sample SWCNT-TPU-5, not enough suitable material for tensile testing could be recovered due to the strong adhesion of the film to the filter membrane. The thermoelectric characterization was carried out in a Seebeck measuring device developed at IPF Dresden as described elsewhere in more detail.^30^ The thermovoltage measurements were performed at 40 °C with a Multimeter Keithley DMM 2001 on the strips (20 × 5 mm^2^) cut from the sample sheets. For each sample, 3–5 cycles were implemented and 2–3 strips per composition were measured. A 4-point measurement configuration combined with the Multimeter Keithley DMM 2001 was used for resistance measurement. In-plane thermal conductivity was measured by the parallel thermal conductance method,^31^ using a previously described test fixture at Dalhousie University (Canada).^32,33^ In this implementation, strips of ∼5 mm width are suspended across a ∼6 mm gap, known heating powers (*P*) are applied to the thermally isolated hot side, and the steady-state temperature difference (Δ*T*) is measured, as a function of the power, with a differential thermocouple. Thermal conductance was calculated as the slope of the power vs Δ*T* and the sample thermal conductivity was determined after measuring and subtracting the background thermal conductance and radiation contribution.^31−33^ These measurements were performed under vacuum conditions (<10^–4^ Torr) at 40 °C.

## 3 Results and Discussion

### 3.1 Morphological Analysis of SWCNT BP and Nonwoven SWCNT–TPU Composite Sheets

Nonwoven SWCNT–TPU composite sheets with a wide range of compositions (Table 1) were produced by applying a one-step filtration method. As reported elsewhere,^26,27^ by changing the concentration of TPU (changing the nanotube/TPU ratio) in an optimized solvent/nonsolvent mixture, SWCNT–TPU sheets with a wide range of compositions can be fast recovered by vacuum filtration. In contrast with using one solvent, an optimized solvent/nonsolvent mixture leads to a strong binding of TPU on the nanotubes as a coating and a significantly lower concentration of TPU in solution that facilitates a fast filtration. In this way, nonwoven SWCNT–TPU composite sheets with tailorable properties are produced. Note that the amount of TPU adsorbed on the nanotubes and appearing in the fabriclike sheets recovered by filtration is lower than the initial amount added in solution, as determined by the partition coefficient of TPU on SWCNTs to the concentration of TPU in the liquid phase. As shown in Table 1, the density of the materials varies with the SWCNT/TPU ratio and generally increases with the TPU content. Using these density values, the SWCNT vol % and void vol % were calculated, and the results are also included in Table 1, revealing their porous nature. Hence, the SWCNT vol % varies from 16 to 3% as the SWCNT/TPU wt % ratio changes from 90/10 to 5/95 for SWCNT-TPU-90 to SWCNT-TPU-5, respectively.

**Figure 2:**
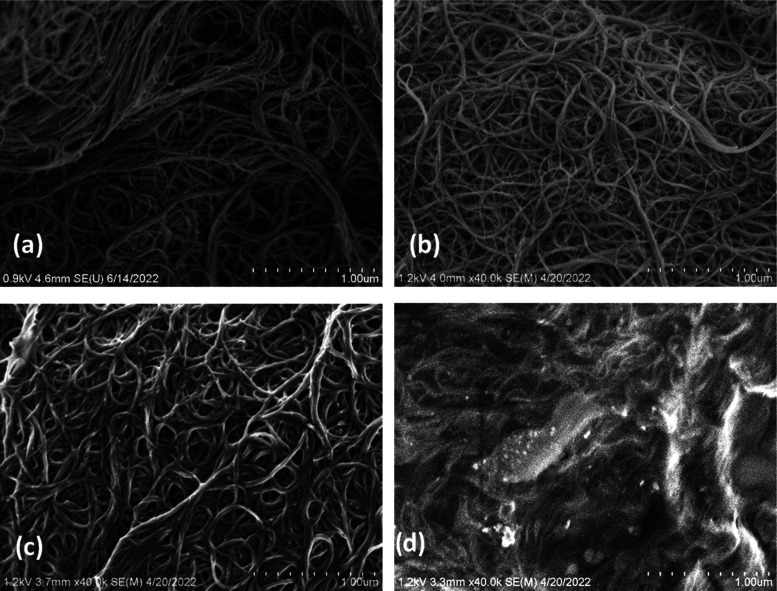
SEM images of the surface of (a) SWCNT BP and (b–d) nonwoven SWCNT–TPU composite sheets of different compositions (Table 1): (b) SWCNT-TPU-90, (c) SWCNT-TPU-35, and (d) SWCNT-TPU-8.

SEM images of the surface of the SWCNT BP and nonwoven SWCNT–TPU composite sheets with different compositions are shown in Figure 2. Additional micrographs and corresponding histograms are included in the SI (Figure S1). For the SWCNT BP (Figure 2a), the characteristic disordered tangle of SWCNT bundles with a porous mesh structure and a wide distribution of bundle diameters (ca. 10–80 nm) was observed. The morphologies of the nanocomposites show similar characteristics (i.e., a “fiberlike” morphology), but significant changes are evident as the SWCNT/TPU ratio changes. The bundle diameters appear to decrease for the sample SWCNT-TPU-90 (Figure 2b) in comparison to the SWCNT BP, showing a narrower distribution (ca. 10–40 nm, Figure S1a), but some larger bundles (ca. 80 nm) are also present. This indicates that the TPU has a favorable interaction with the nanotubes while still highly dispersed in solution and limits rebundling during formation of the sheets, which results in smaller bundle sizes. As shown in Figures 2c and S1b, with increasing TPU content (Table 1), the bundle diameters of the samples SWCNT-TPU-50 (SWCNT/TPU ratio of 50/50) and SWCNT-TPU-35 (SWCNT/TPU ratio of 35/65) are in the range of 20–40 nm and the polymer is clearly observed to be coating the nanotubes/bundles. Hence, the observed bundle diameter is determined not only by the SWCNT bundle diameter but also by the increasing amount of TPU coating. Voids are also visible on the surface, which is consistent with the low density measured for these sheets (Table 1). As shown in Figure 2d for SWCNT-TPU-8, the surface morphology of samples with a significantly higher content of TPU (SWCNT/TPU ratio of 8/92) is dominated by a surface layer of TPU while embedded nanotubes are visible under the rough surface. Similar to MWCNT–TPU sheets,^26^ the morphological analysis and physical characterization of the SWCNT–TPU sheets (Table 1) show that TPU forms a coating on the SWCNTs. As the TPU content increases and the available surface area is saturated, additional or excess TPU deposits in combination with TPU-coated nanotubes and the sheet thickness significantly increases. This additional TPU leads to morphological changes, where the porous mesh structure is no longer visible.

### 3.2 Mechanical Properties of SWCNT BP and Nonwoven SWCNT–TPU Composite Sheets

**Figure 3:**
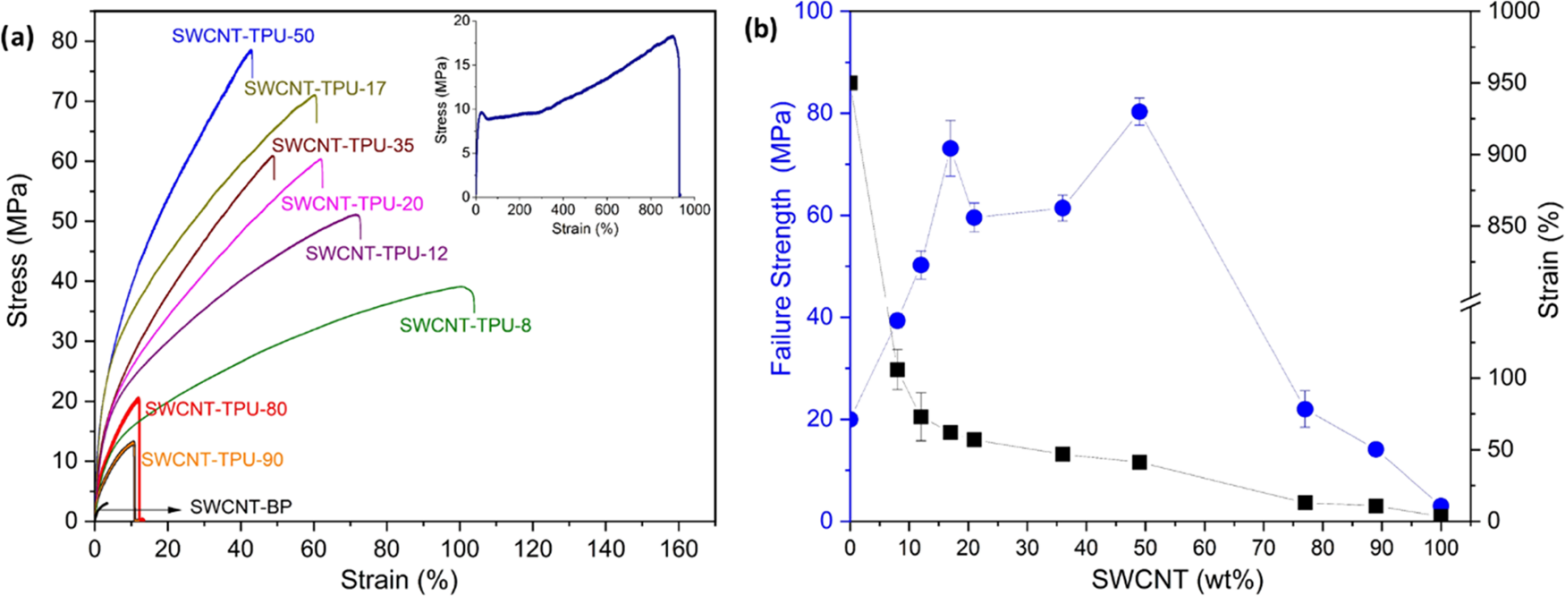
(a) Representative stress–strain curves for nonwoven SWCNT–TPU composite sheets of varying composition, SWCNT BP, and pristine TPU (inset). (b) Average failure strength and strain for the SWCNT–TPU composite sheets, SWCNT BP, and pristine TPU (lines to guide the eye).

The tensile properties are summarized in Table 2 and Figure 3. Figure 3a shows representative stress–strain curves for sheets of different compositions together with pristine TPU and a SWCNT BP. The characteristic behavior of pristine TPU is observed (Figure 3a inset), including the region of plastic flow deformations and the strain-hardening region, achieving a failure strength (σ_fail_) of ∼20 MPa. The behavior observed for the nonwoven SWCNT–TPU composite sheets differs from that of TPU, without the pronounced strain-hardening effect. In comparison to the pristine SWCNT BP, as the TPU content increases, the failure strength increases and it reaches the highest values in the range of 50 to 17 wt % of SWCNTs (50/50 to 17/83 wt % ratios) before decreasing substantially at the highest TPU contents. The Young’s modulus (*E*) shows similar trends, while the failure strain (ε_fail_) shows the typical behavior observed for this type of nanocomposite (i.e., reduction in ε_fail_ as the nanotube content increases). Consequently, the tensile toughness of the nanocomposites (*G*_t_) is lower than for pristine TPU. However, a combination of significantly improved strength and stiffness for the SWCNT–TPU sheets can lead to higher absorbed energy per volume at an equivalent strain (e.g., *G*_ε=40%_, see Table 2).

**Table 2:** Mechanical Tensile Properties of the SWCNT BP, Pristine TPU, and Nonwoven SWCNT–TPU Composite Sheets

sample	*E* (MPa)	σ_fail_ (MPa)	ε_fail_ (%)	*G*_t_ (MJ/m^3^)	*G*_ε=40%_ (MJ/m^3^)
SWCNT BP	270 ± 30	3 ± 0.4	3.3 ± 0.7	0.09 ± 0.03	
SWCNT-TPU-90	576 ± 75	14 ± 1	11 ± 1	1.1 ± 0.1	
SWCNT-TPU-80	955 ± 81	22 ± 4	13 ± 2	2 ± 0.6	
SWCNT-TPU-50	1773 ± 91	80 ± 3	41 ± 1	23 ± 1	21 ± 0.9
SWCNT-TPU-35	1068 ± 57	61 ± 2	47 ± 14	19 ± 4	14 ± 2
SWCNT-TPU-20	839 ± 31	60 ± 3	57 ± 5	23 ± 3	13 ± 0.5
SWCNT-TPU-17	1517 ± 117	73 ± 5	62 ± 4	33 ± 5	17 ± 0.8
SWCNT-TPU-12	828 ± 72	50 ± 3	73 ± 17	27 ± 7	11 ± 0.3
SWCNT-TPU-8	469 ± 28	39 ± 1	106 ± 14	31 ± 5	7 ± 0.3
TPU	160 ± 14	18 ± 2	764 ± 126	94 ± 20	3 ± 0.1

Similar trends in mechanical properties were also observed in our previous study with MWCNT–TPU sheets^26^ and can be explained as a result of the morphological changes occurring as a function of decreasing the MWCNT/TPU ratio (increasingly adding TPU to the nanotube suspension). At a certain composition, a better MWCNT debundling/disentanglement and optimal CNT surface coverage by TPU were achieved (optimized MWCNT/TPU interface), which in turn led to an optimal packing of MWCNT/TPU fibers, and the highest improvement in σ_fail_ and E. Above and below this composition, the mechanical properties decreased. For SWCNT–TPU sheets produced here, the highest enhancements are observed over a wider range of compositions from 50 to 17 wt % content of SWCNTs (vs ∼35 wt % for MWCNT–TPU^26^). We attribute this behavior to the acetone/methanol solvent system being less efficient at achieving optimal SWCNT debundling/disentanglement and optimal packing in the obtained SWCNT–TPU sheets. SWCNTs, with their smaller diameters, form more closely packed bundles with the van der Waals forces holding them together more strongly compared to MWCNTs and require a better solvent (e.g., dimethylformamide) to improve their debundling/disentanglement, which is expected to be more effective to expose a high SWCNT surface for TPU adsorption. A nonoptimal debundling in methanol could have led to a nonoptimal SWCNT surface coverage by TPU and SWCNT–TPU fiber packing as the TPU content increased (from 50 to 17 wt % content of SWCNTs). This may have resulted in similar strength values in a wider composition range rather than a clear maximum in a defined or narrower composition range. Also, it is worth noting that the thickness of the SWCNT-TPU-17 sample is significantly lower than that of SWCNT-TPU-20 despite containing a higher TPU content, indicating a better network packing and the complex effect of SWCNT/TPU ratio on the characteristics of the nanocomposite network. Nevertheless, due to the higher aspect ratio and specific surface area of SWCNTs in comparison to MWCNTs, the mechanical properties are significantly improved. The highest improvements are achieved for the sample SWCNT-TPU-50 at the 50/50 SWCNT/TPU weight ratio (SWCNT 15 vol %), with 2- and 4-fold improvement in σ_fail_ compared to similarly prepared MWCNT–TPU sheets^26^ and to pristine TPU, respectively. Moreover, due to the porous nature of these materials, they all have densities ranging from 0.31 to 1.02 g cm^–3^, which are below the density of TPU (1.19 g cm^–3^); hence, their specific properties are further enhanced as compared to TPU.

### 3.3 Thermal Conductivities of SWCNT BP and Nonwoven SWCNT–TPU Composite Sheets

**Figure 4:**
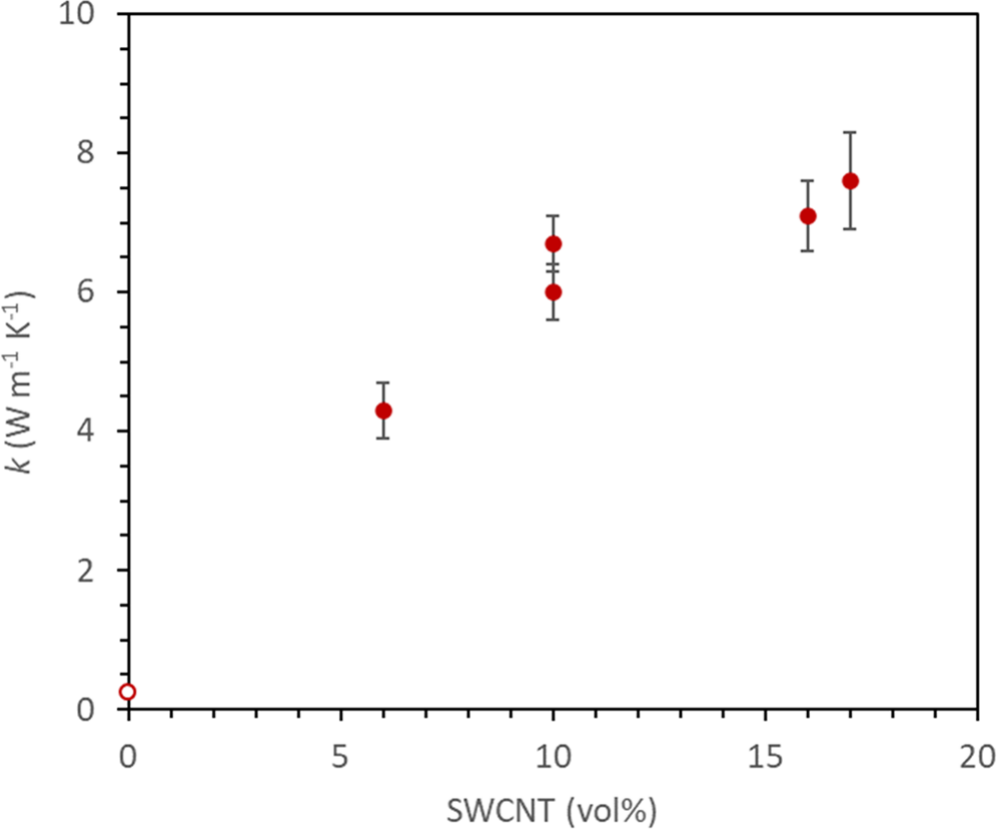
(●) Measured thermal conductivity of nonwoven SWCNT–TPU composite sheets vs the vol % of SWCNTs. (○) Thermal conductivity of unfilled TPUs.

The thermal conductivities of samples with compositions near 5, 10, and 15 vol % SWCNTs, which were selected to cover the majority of the compositional range, are shown in Figure 4. Thermal conductivity ranged from 4.3 to 7.6 W m^–1^ K^–1^, much higher than typical values (e.g., 0.20 W m^–1^ K^–1^)^14^ for TPUs, and was expected to depend primarily on the volume fraction of SWCNTs. The SWCNT–TPU sheets more closely resemble nonwoven fabrics rather than fully dense composites, in particular for moderate to high SWCNT contents (∼20–90 wt % SWCNTs). In addition to the SWCNT content, the porosity (void vol %) also varies with the weight ratio of SWCNT to TPU. For example, samples with 50 and 90 wt % SWCNTs all yield ∼15 vol % SWCNTs due to differences in packing and porosity. The TPU can affect the SWCNT network thermal conductivity both positively, by improving SWCNT dispersion (SWCNT debundling/disentanglement in solution) and hence packing (SWCNT–TPU “fibers” entanglement) in the recovered sheets, and negatively, due to the presence of a polymer layer at CNT junctions, and TPU itself also contributes to heat conduction to the extent that the polymer component would otherwise be unfilled void space. Empirically, a linear trend (*R*^2^ = 0.99) was observed for thermal conductivity normalized to density as a function of wt % nanotubes (Figure S2a), which indirectly considers the effect of TPU. From this, the thermal conductivities of the compositions that were not measured can be estimated (Figure S2b) in order to calculate *ZT*.

The effect of TPU on the measured thermal conductivity of the composite (*k*_comp_) can also be interpreted using a simple rule-of-mixture model based on addition of thermal resistances in parallel, i.e.
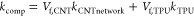
1where *V*_f*,i*_ is the volume fraction of component *i* (*i* = CNT network, TPU, void) and the contribution of voids is negligible as the measurements are performed under high vacuum. Implicitly, the *k*_CNTnetwork_ term not only incorporates the thermal conductivity of the CNTs (i.e., *k*_SWCNT_) but also includes effects of CNT bundling and interfacial thermal resistances (i.e., between CNTs or CNT bundles). With *k*_comp_ determined by the thermal conductivity measurement, the thermal conductivity of the SWCNT network can be estimated as
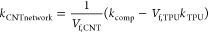
2The thermal conductivity of TPU is relatively low, which makes the *V*_f,TPU_*k*_TPU_ term small (<5% effect on *k*_CNTnetwork_ for the high TPU content SWCNT-TPU-12 sample) to negligible (1% effect on *k*_CNTnetwork_ and lower for SWCNT-TPU-35 and higher SWCNT content samples). This analysis proves that the direct contribution of TPU to the heat transfer in these composites is minimal (i.e., *k*_comp_ ≈ *V*_f,CNT_*k*_CNTnetwork_); however, as shown in Figure 4, thermal conductivity deviates from a linear trend with *V*_f,CNT_. Therefore, the presence of TPU in the processing must lead to a more thermally conductive nanotube network (i.e., a higher *k*_CNTnetwork_). From eq 2, *k*_CNTnetwork_ increases from ∼45 W m^–1^ K^–1^ for SWCNT BP and SWCNT-TPU-90 to ∼60 W m^–1^ K^–1^ with moderate TPU content (SWCNT-TPU-35) and 65–70 W m^–1^ K^–1^ for the highest TPU content composites measured (i.e., SWCNT-TPU-17 and SWCNT-TPU-12, respectively).

### 3.4 Thermoelectric Properties of SWCNT BP and Nonwoven SWCNT–TPU Composite Sheets

**Figure 5:**
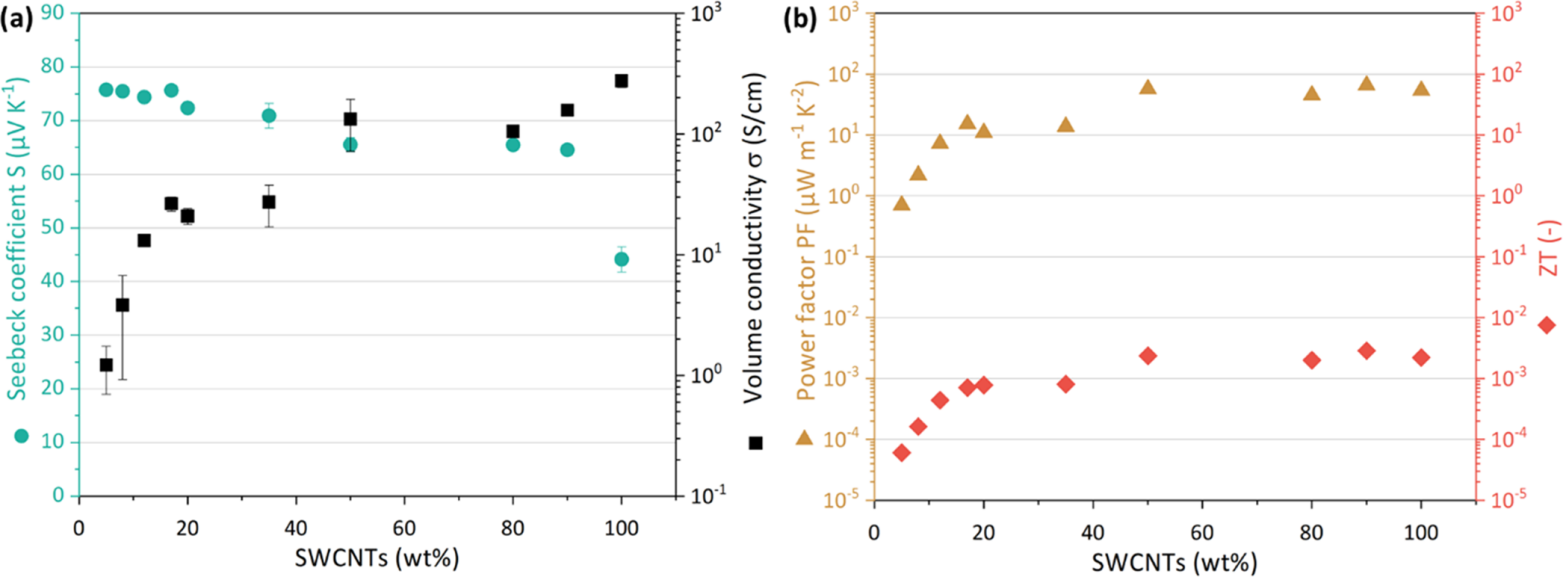
Thermoelectric properties of nonwoven SWCNT–TPU composite sheets and SWCNT BP. (a) σ and S, and (b) average PF and *ZT*.

The σ and S, power factor, and figure of merit at 40 °C for the SWCNT BP and SWCNT–TPU composite sheets are presented in Figure 5 and the SI (Table S1 and Figure S3). The SWCNT BP shows an in-plane σ of 276 ± 33 S cm^–1^. This σ is lower than the 422 S cm^–1^ reported for BPs of the same SWCNTs prepared using chloroform (CF).^28^ An interesting study also reports higher σ in the range of 558–893 S cm^–1^ for BPs of SWCNTs (Tuball) prepared using ethanol (EtOH), methanol (MeOH), acetone (AC), tetrahydrofuran (THF), and acetonitrile (ACN).^34^ As expected, the σ values of TPU–SWCNT composites are lower than those of the BP (Table S1). The encasing of TPU around the SWCNTs must increase the electrical contact resistance between the adjacent SWCNTs, which explains the decrease of the electrical conductivity in the SWCNT–TPU sheets.^35^ In particular, the electrical conductivity increased as a function of SWCNT weight content from 1.2 ± 0.5 S cm^–1^ (SWCNT-TPU-5) to 157 ± 17 S cm^–1^ (SWCNT-TPU-90). As expected, the σ value reported here for SWCNT-TPU-90 is the highest among TPU–CNT composites, which is due to the high loading of SWCNTs achieved by this novel method of preparation (see Table S2). For instance, a lower value of 110 S cm^–1^ has been reported for self-supporting films prepared by drop-casting using 10 wt % SWCNT Tuball wrapped around polymer particles and a water-dispersible polyurethane with blocked terminal isocyanate groups (PUBI).^13^

The Seebeck coefficients of the SWCNT BP and SWCNT–TPU composite sheets at 40 °C are also presented in Figure 5a and the SI (Table S1 and Figure S3). The SWCNT buckypaper shows an *S* of 44.1 ± 2.3 μV K^–1^ somewhat higher than the value of 37.4 μV K^–1^ found in BPs made with the same SWCNTs (Tuball) mentioned before.^28^ However, higher S (from 48.2 to 59.1 μV K^–1^ for BPs of SWCNTs (Tuball) prepared with EtOH, MeOH, AC, THF, or ACN) or lower *S* (16.7 μV K^–1^ with CF as the solvent) has been already reported.^34^ As expected, the SWCNTs utilized in this work show p-type character as most of commercial as-produced CNTs.^3^ Accordingly, SWCNT–TPU composite sheets also show positive *S* values (Table S1). Moreover, the Seebeck coefficients found for all SWCNT–TPU composites in this study are higher than those reported recently for comparable materials (Table S2). In contrast to σ, *S* decreases with increasing SWCNT weight percent content. In particular, the Seebeck coefficient increased from 64.5 ± 0.3 μV K^–1^ (SWCNT-TPU-90) to 75.7 ± 0.1 μV K^–1^ (SWCNT-TPU-5). This trend matches well with the typical behavior of heterogeneous conducting polymer composites, where an increase in *S* with decreasing σ is attributed to the inverse σ-dependence of the energy barrier term in the thermal fluctuation-induced tunneling model.^15^ Nevertheless, the *S* value of the BP, which should represent a composite sheet of 0 wt % TPU and 100 wt % SWCNT, does not fit this trend. This is reflected in the considerable jump of *S* observed between the CNT-TPU-90 composite sheet (64.3 μV K^–1^) and the BP (44.1 μV K^–1^). This discrepancy means that the TPU, despite its insulating character, alters the p-type behavior of the SWCNTs, which is seen in earlier studies that show that the Fermi energy level of the graphene layers of SWCNTs can be influenced by surrounding polymer molecules acting as dopants.^36^

Raman spectroscopy is a frequently used method to probe SWCNT materials and composites due to the resonant nature of the Raman excitation and commensurate sensitivity to effects such as doping, surface chemistry, and mechanical strain.^37,38^Figure S4 shows Raman spectra obtained for a SWCNT buckypaper and nanocomposite sheets of different compositions, where the typical bands for SWCNTs are observed.^39^ Changes to the position and peak profile of the G-band (∼1590 cm^–1^) can be indicative of doping in SWCNTs.^38,40,41^ In these samples, we observe no differences in the G-band between the buckypaper and the composite samples, suggestive of there being no significant change in the nanotube electronic structure upon forming the TPU composites. This is not inconsistent with previous works that showed that stronger doping resulted in only small shifts of the G-band position (∼1–3 cm^–1^),^41^ which has been attributed to pristine SWCNTs in ambient conditions already having p-doped character due to adsorbed oxygen.^40,41^ The intensity ratio between the D-band (∼1350 cm^–1^) and G-band is used to assess disruptions to the sp^2^ framework of the SWCNTs, such as from the introduction of defects or chemical bonds on the nanotube walls. Again, the observed D/G ratios were also very similar across these samples (Figure S4d). The radial breathing modes (RBMs) of SWCNTs are even more sensitive to changes in surface chemical and charge states, though the precise interpretation can be challenging due to differences in excitation profiles and individual chirality nanotubes within the ensemble moving in and out of resonance with the Raman excitation laser as a result of sample modifications.^38,42^ In the RBM region (Figure S4c,f), we observe some variability in the peak positions and intensities within multiple measurements of each sample composition, including for the pristine buckypaper. We assume that this is attributable to slight inhomogeneities across the samples (e.g., differences in TPU surface coverage and thickness in the composites, and differences in bundling and adsorbates for the buckypaper). Nevertheless, we do observe some small but consistent differences between the RBM regions of the buckypaper and the composites (Figure S4c,f), most notable being that the ∼265 cm^–1^ peak observed in the buckypaper is absent in the composites and sometimes replaced with a new peak at ∼245 cm^–1^. In consulting the Kataura plot,^42^ we can see that peaks in this region are derived from the SWCNTs at the very smallest end of the diameter distribution for Tuball nanotubes, and for which there are a very small number of possible chiralities that would be in resonance with the Raman excitation laser (i.e., 2.41 eV). This subset of the nanotube population at the extreme end of the diameter distribution would be most sensitive to surface changes, so it is not surprising that we observe some of these nanotubes moving in and out of resonance with the laser upon forming the composites due to polymer–SWCNT interactions.

The power factors at 40 °C of the SWCNT–TPU composite sheets and SWCNT BP were calculated (Table S1) and presented in Figure 5b. In particular, the SWCNT BP shows a PF of 53.7 μW·m^–1^·K^–2^, which is lower than 59.2^28^ and 64.0 μW·m^–1^·K^–2^ reported for SWCNT (Tuball) BP prepared with CF.^34^ A superior PF of 266 μW·m^–1^·K^–2^ has been reported for SWCNT (Tuball) BPs prepared with EtOH.^34^ Therefore, this study confirms that the thermoelectric properties of BPs with the same type of SWCNTs (Tuball) are affected not only by the preparation method but also by the solvent utilized in the preparation of the SWCNT dispersions. In addition, it is noticed that for SWCNT–TPU sheets, the highest PF (65.4 μW·m^–1^·K^–2^) was found in the SWCNT-TPU-90 sample, which is larger than the PF values reported recently for similar materials (Table S2). The PF decreases only slightly from the 90 to 50 wt % SWCNT composition, before substantially decreasing for 35 wt % SWCNT. A similar trend was observed for the electrical conductivity and can be explained by the corresponding changes in SWCNT vol % (Table 1 and Figure S3). The SWCNT vol % remains almost constant (16–15 vol %) as the TPU content increases to the 50/50 wt % ratio composition before decreasing to 10 vol % in the SWCNT-TPU-35 sample. The complex morphological changes that occur as a function of increasingly covering SWCNTs with TPU impact the characteristics of the SWCNT network and the properties of the SWCNT–TPU sheets.

The figure of merit  at 40 °C of the SWCNT–TPU composite sheets was found to reach values of *ZT* 2.9 × 10^–3^ and 2.4 × 10^–3^ for SWCNT contents of 90 wt % (16 vol % SWCNTs) and 50 wt % (15 vol % SWCNTs), respectively. To the best of the authors′ knowledge, these are the highest *ZT* values reported for TE materials consisting of TPU and carbon-based materials only. As reference, a higher *ZT* of 4.1 × 10^–1^ has been estimated for composites consisting of insulating polystyrene and 75 wt % of SWCNT fabricated by a planetary ball-milling-based dispersion technique.^43^ However, as pointed out by the authors, the *k* values in the through-thickness direction were used for estimating the *ZT* values, which are expected to vary from the actual *ZT* because *k* of the composite exhibited strong anisotropy. As shown in Figure 5b and Table S1, the figure of merit follows the same trend as the power factor. This is consistent with expectations for CNT–polymer composites, as the thermal conductivity changes only modestly over most of the composition range.

In summary, given that the mechanical properties are also significantly better than those of the samples with higher SWCNT content (including the SWCNT BP), we can conclude that the nonwoven SWCNT–TPU composite sheet with 50 wt % of SWCNTs (15 vol % SWCNTs) is the preferred option for further development of a flexible TE module as the p-type component. The need to improve the mechanical properties of CPCs without sacrificing their TE performance has received increased attention,^44^ and high flexibility, strength, and toughness are essential for reliable wearable TE devices to withstand textile manufacturing and body movement.^45^ The fabrication method employed here allows for the optimization of thermoelectric and mechanical properties. The approach has also been shown to be scalable and to provide a range of other multifunctional properties including flame resistance,^46^ making it attractive for large-area thermoelectric devices that are needed to harvest the large amount of thermal energy available at low temperatures.^47^

## 4 Conclusions

In the field of flexible electronics, the development of advanced materials should be associated with the use of strategic materials and methods to reduce costs without compromising mechanical and electrical performance. Here, we combined a thermoplastic polyurethane (TPU) with commercial single-wall carbon nanotubes (SWCNTs) to fabricate SWCNT–TPU self-supporting composite sheets with a good combination of thermoelectric properties and mechanical robustness. By employing a versatile one-step filtration method, the full scope of the SWCNT/TPU ratio on the SWCNT surface coverage by TPU, disentanglement, and packing was analyzed through the evaluation of mechanical and thermoelectric properties. The incorporation of a small amount of TPU (SWCNT-TPU-90 with a 90/10 SWCNT/TPU wt % ratio) significantly decreases the electrical conductivity and increases the Seebeck coefficient. However, in the 90/10 to 50/50 wt % ratio range, the increased amount of the insulating TPU is not detrimental to the electrical transport of the nanocomposite network, the Seebeck coefficient and thermal conductivity are almost constant, and the mechanical properties are significantly increased. These results are explained by the complex morphological changes that occur as a function of increasingly covering SWCNTs with TPU. In particular, a 50 wt % (SWCNT 15 vol %) nanocomposite sheet with a high Young’s modulus of 1.8 GPa, failure strength of 80 MPa, elongation at break of 41%, high electrical conductivity of 133 S cm^–1^, Seebeck coefficient of 65 μV K^–1^, and power factor of 57 μW m^–1^ K^–2^ corresponding to a *ZT* of 2.4 × 10^–3^ is achieved. TPU is shown to have a positive effect at improving the SWCNT debundling/disentanglement in solution that leads to a better network packing as well as possibly p-doping. Consisting of commercial materials combined in a single step, the present approach represents a feasible route to obtain flexible and stretchable p-type components for energy-harvesting modules based on the thermoelectric effect.

## Supporting Information Available

Additional SEM images of the BP and nanocomposite sheets; diameter size histograms of SWCNT/TPU bundles; thermal conductivity and thermoelectric property data; comparative table with thermoelectric properties of carbon nanotube/TPU based composites; and experimental details (PDF)
