# Supplementary material for: Carbon Nanotube–Polyurethane Composite Sheets for Flexible Thermoelectric Materials
Source: ACS Appl Nano Mater. 2023 Sep 19;6(19):17986–95. doi: 10.1021/acsanm.3c03247 (PMC10580240; doi:10.1021/acsanm.3c03247)
Supplement: Supplementary file 1 — an3c03247_si_001.pdf [file an3c03247_si_001.pdf]

Supporting Information

Carbon Nanotube-Polyurethane Composite Sheets for Flexible Thermoelectric Materials

Antonio J. Paleo[1], Yadienka Martinez-Rubi[2*], Beate Krause[3], Petra Pötschke[3], Michael B. Jakubinek[2], Behnam Ashrafi[4] and Christopher Kingston[2]

[1]2C2T-Centre for Textile Science and Technology, University of Minho, 4800-058 Guimarães, Portugal

[2]Security and Disruptive Technologies Research Centre, National Research Council Canada, Ottawa, Ontario K1A 0R6, Canada

[3]Leibniz-Institut für Polymerforschung Dresden e.V. (IPF), Hohe Str. 6, 01069 Dresden, Germany

[4]Aerospace Research Centre, National Research Council Canada, 5145 Decelles Avenue, Montreal, Quebec H3T 2B2, Canada

[*]Yadienka.Martinez-Rubi@nrc-cnrc.gc.ca

2.2 Preparation of SWCNT BP and nonwoven SWCNT-TPU composite sheets

By using a 50/50 ratio of solvent/nonsolvent (equal volumes of the solvent and nonsolvent), TPU that was initially dissolved in acetone is adsorbed onto the surface of SWCNTs upon combination with the non-solvent (methanol), and forms a TPU coating on the nanotubes. The composition (i.e., SWCNT and TPU content) was evaluated by weighing the dried sheets to determine the amount of TPU adsorbed/deposited on the known initial mass of SWCNTs. The external volume of the samples was evaluated by cutting squares of 15 cm × 15 mm from the SWCNT-TPU sheets. The thickness was measured with a Marathon Digital Electronic Micrometer having a resolution of 0.001 mm. The volume fraction of SWCNTs ($V_{f,CNT}$), TPU ($V_{f,TPU}$) and pores/voids ($V_{f,voids}$) in the samples were estimated using equations 1-3:

$$V_{f,CNT} = \frac{\rho_{Comp}}{\rho_{CNT}} W_{f,CNT}, \qquad (1)$$

$$V_{f,TPU} = \frac{\rho_{Comp}}{\rho_{TPU}} W_{f,TPU}, \qquad (2)$$

$$V_{f,voids} = 1 - V_{f,TPU} - V_{f,CNT}, \quad (3)$$

Where ρ_{Comp}, ρ_{CNT} and ρ_{TPU} are the densities of the nanocomposite sheet, SWCNTs, TPU, respectively. ρ_{Comp} was obtained by dividing the mass of the SWCNT–TPU sheets by their external volume, while a literature value of 1.8 g cm^{-3} value was used for ρ_{CNT} [1]. $W_{f,CNT}$ and $W_{f,TPU}$ correspond to the weight fraction of SWCNTs and TPU in the SWCNT-TPU composite sheets, respectively.

3.1 Morphological analysis of SWCNT BP and nonwoven SWCNT-TPU composite sheets

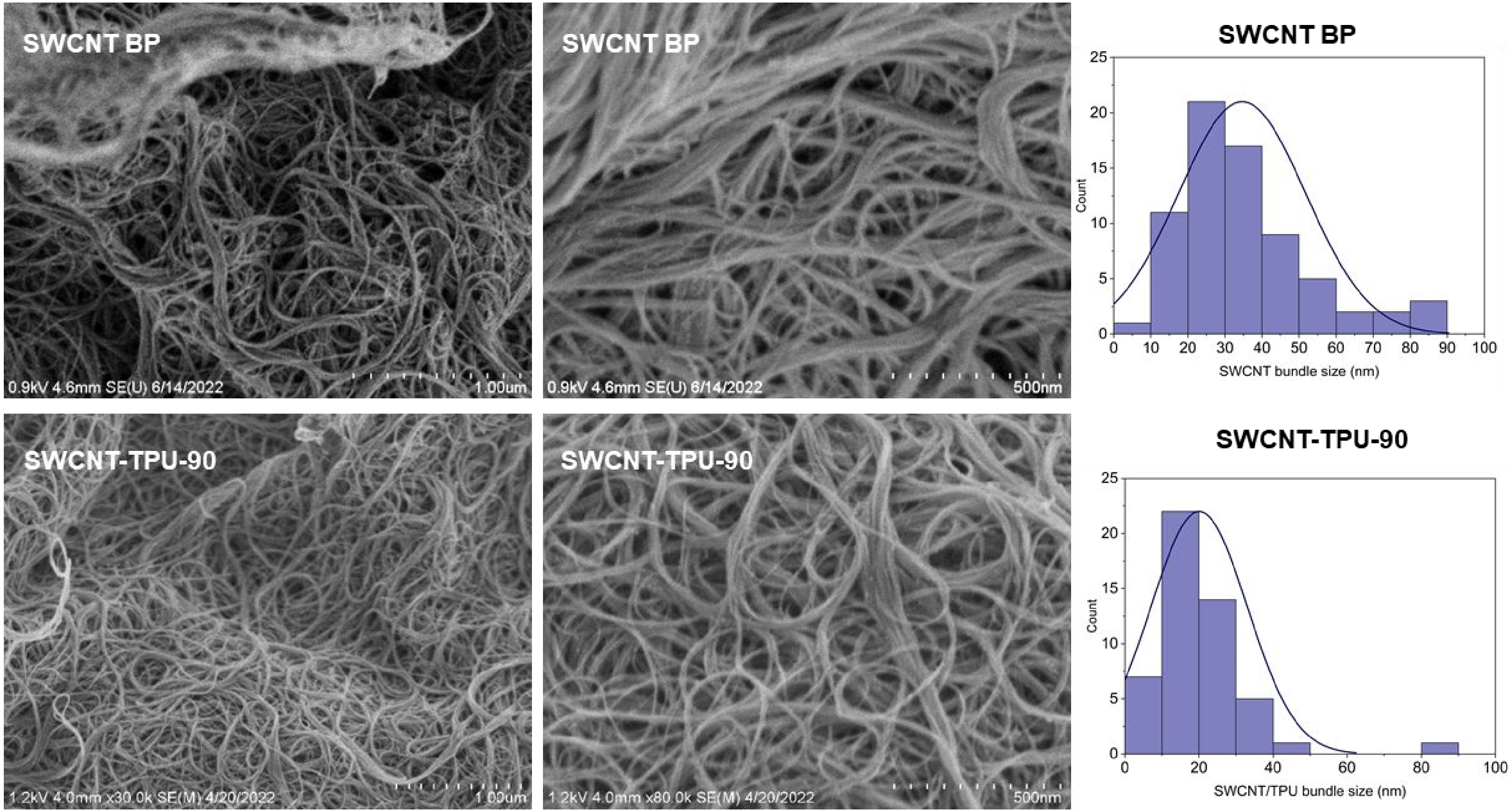

Figure S1a. SEM images of the surface of SWCNT BP and nonwoven SWCNT-TPU-90 composite sheet and corresponding diameter size histograms of SWCNT and SWCNT/TPU bundles

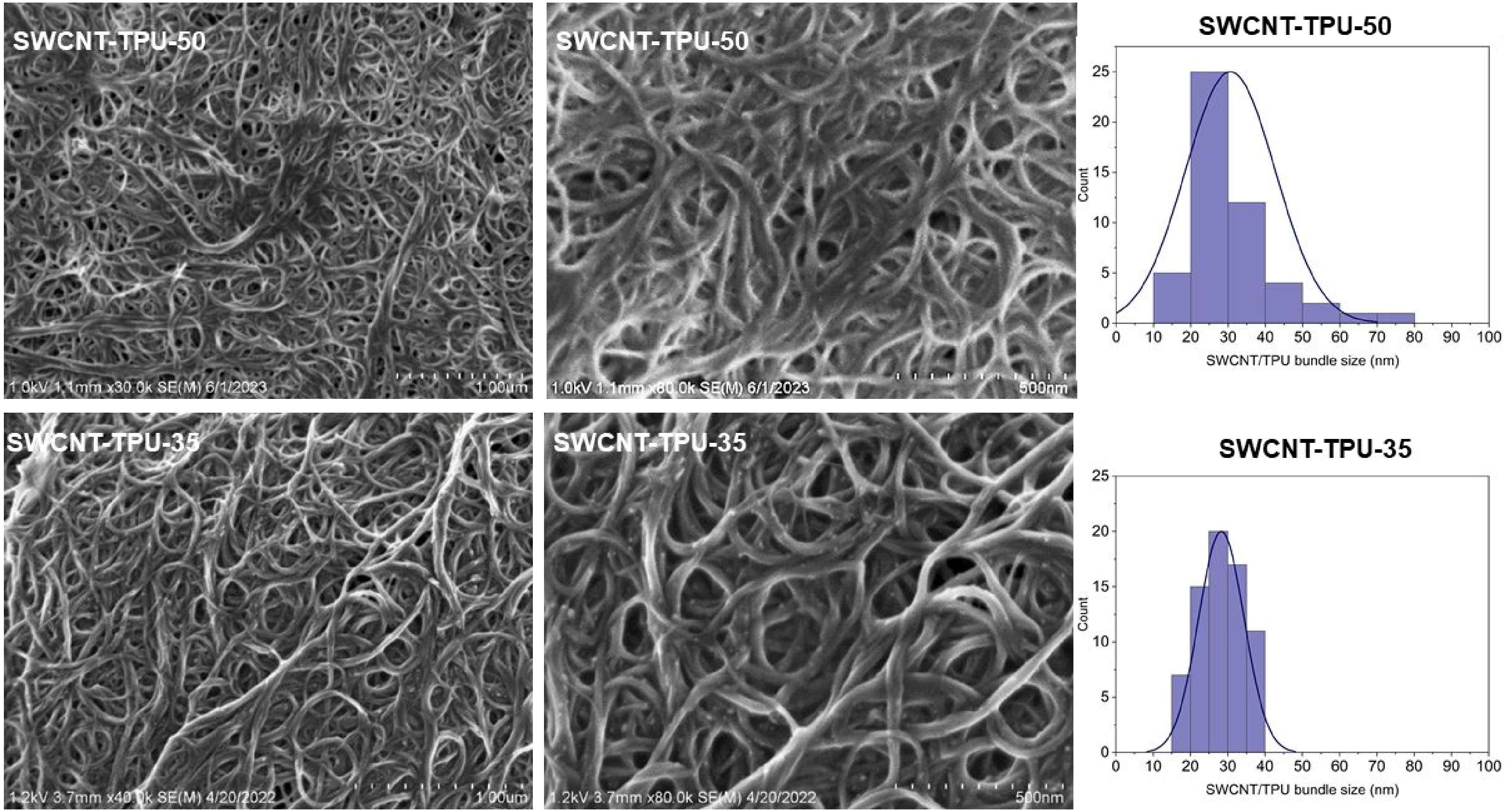

Figure S1b. SEM images of the surface of nonwoven SWCNT-TPU composite sheets with different SWCNT contents and corresponding diameter size histograms of SWCNT/TPU bundles

3.3 Thermal conductivity

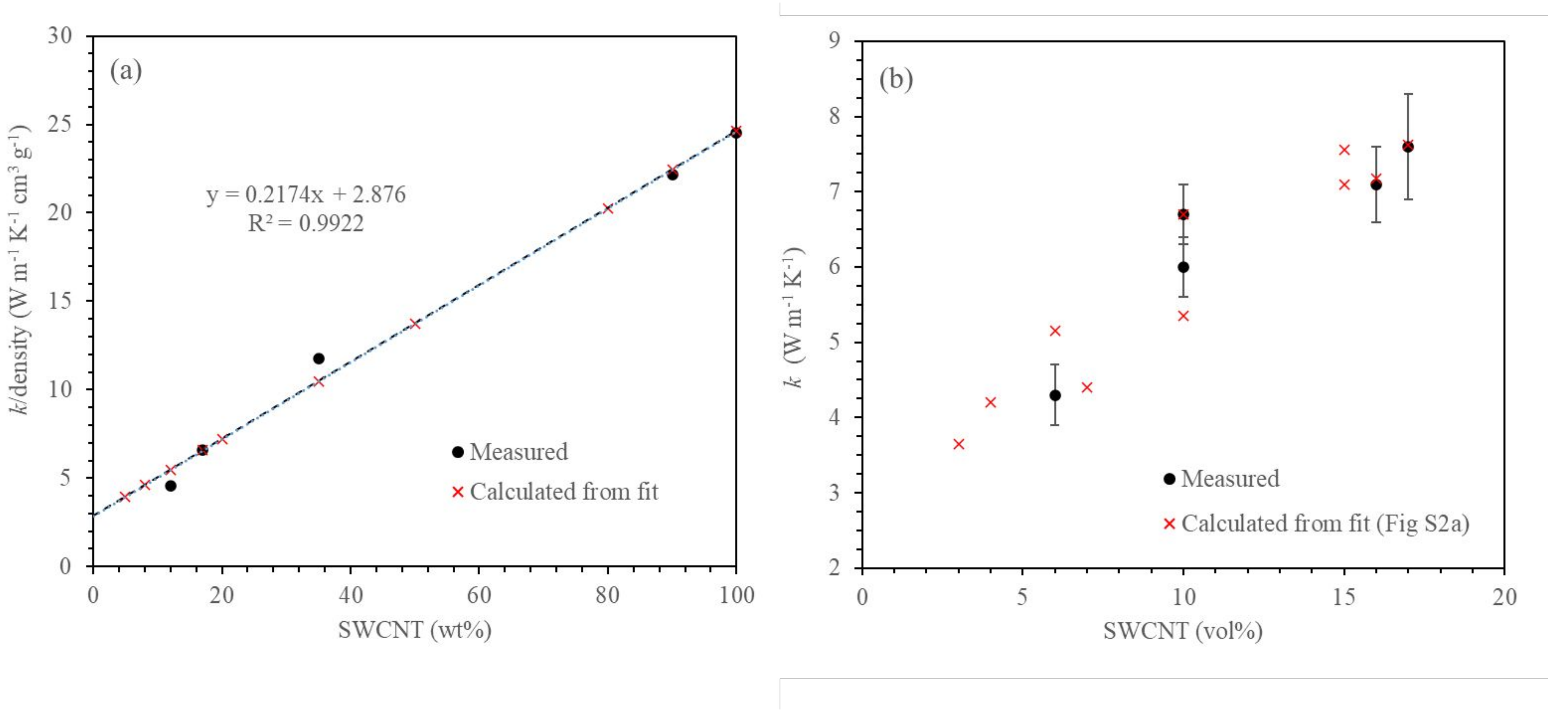

Figure S2. (a) Thermal conductivity (*k*) normalized to density and (b) *k* of the composite samples as a function of vol % SWCNTs including measured datapoints (per Figure 4 in the main text) and estimated data from the linear fit.

3.4 Thermoelectric properties of SWCNT BP and nonwoven SWCNT-TPU composite sheets

Table S1. Electrical conductivity *σ*, Seebeck coefficient *S*, power factor PF, thermal conductivity *k*, and figure of merit ZT at 40 °C of the investigated samples.

Sample	*σ* (S cm^{-1})	*S* (µV K^{-1})	Average PF (µW $m^{-1}·K^{-2}$)	*k* (W m^{-1} K^{-1})	Average ZT (-)
SWCNT BP	276 ± 33	44.1 ± 2.3	53.7	7.6 ± 0.7	2.2×10^{-3}
SWCNT-TPU-90	157 ± 17	64.5 ± 0.3	65.4	7.1 ± 0.5	2.9×10^{-3}
SWCNT-TPU-80	105 ± 5	65.5 ± 0.5	45.0	*	2.0×10^{-3}
SWCNT-TPU-50	133 ± 61	65.5 ± 1.3	56.9	*	2.4×10^{-3}
SWCNT-TPU-35	27 ± 10	70.9 ± 2.3	13.8	6.0 ± 0.4	0.7×10^{-3}
SWCNT-TPU-20	21 ± 3	72.3 ± 0.4	10.9	*	0.8×10^{-3}
SWCNT-TPU-17	27 ± 4	75.6 ± 0.7	15.2	6.7 ± 0.4	0.7×10^{-3}
SWCNT-TPU-12	13 ± 1	74.4 ± 0.0	7.2	4.3 ± 0.4	0.5×10^{-3}
SWCNT-TPU-8	3.8 ± 2.9	75.5 ± 0.9	2.2	*	0.2×10^{-3}
SWCNT-TPU-5	1.2 ± 0.5	75.7 ± 0.1	0.7	*	0.06×10^{-3}

*For these composites, the thermal conductivity value was not measured, instead an interpolated value based on Figure S2b was used. Given the modest variation in *k*, this is not expected to have a major effect on the estimated ZT values and trends.

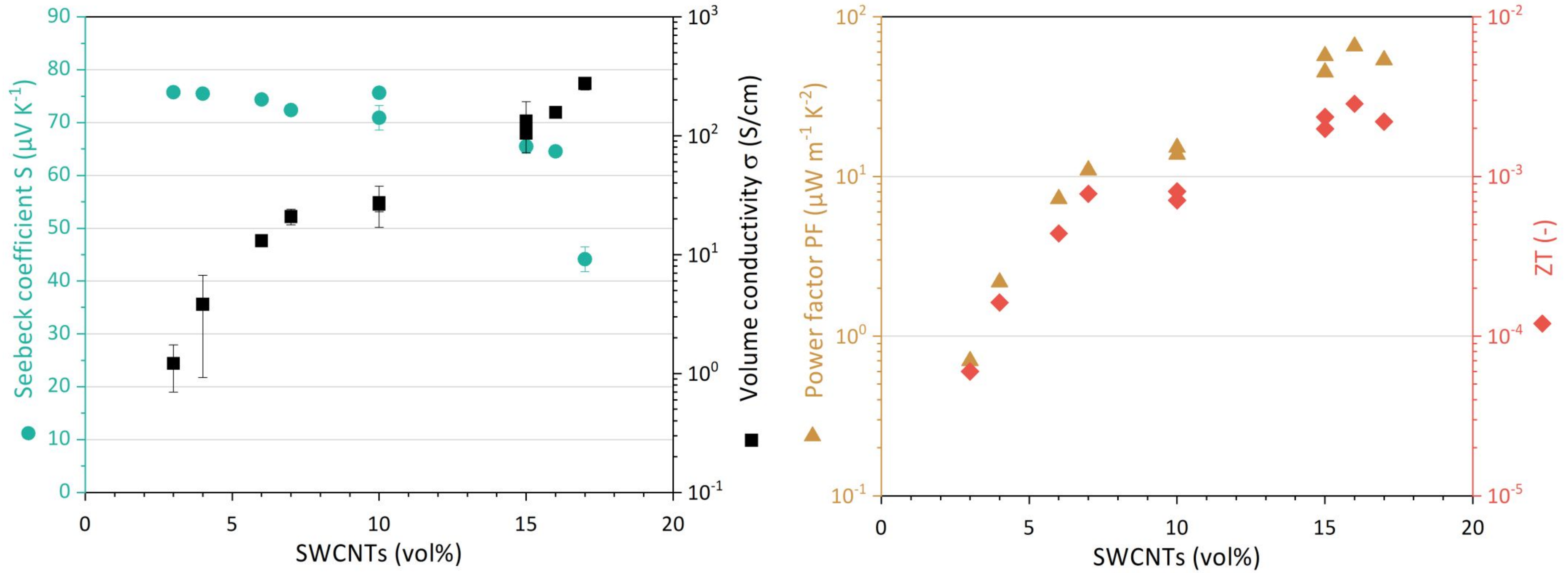

Figure S3. Thermoelectric properties of nonwoven SWCNT-TPU composite sheets and SWCNT BP as a function of the SWCNT vol %

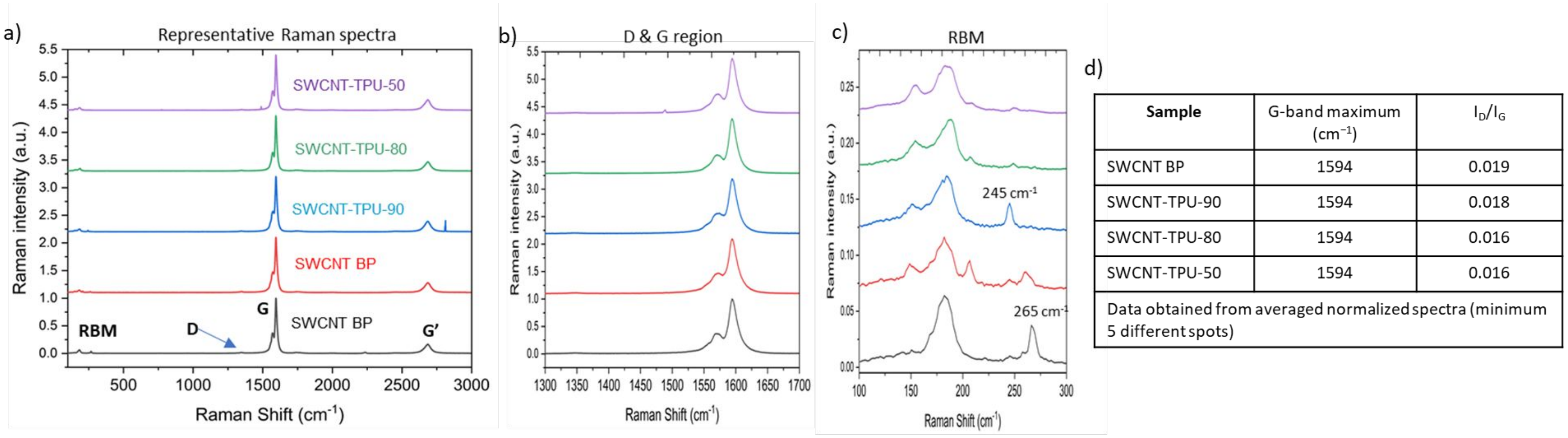

d)

Sample	G-band maximum (cm^{-1})	I_D/I_G
SWCNT BP	1594	0.019
SWCNT-TPU-90	1594	0.018
SWCNT-TPU-80	1594	0.016
SWCNT-TPU-50	1594	0.016
Data obtained from averaged normalized spectra (minimum 5 different spots)		

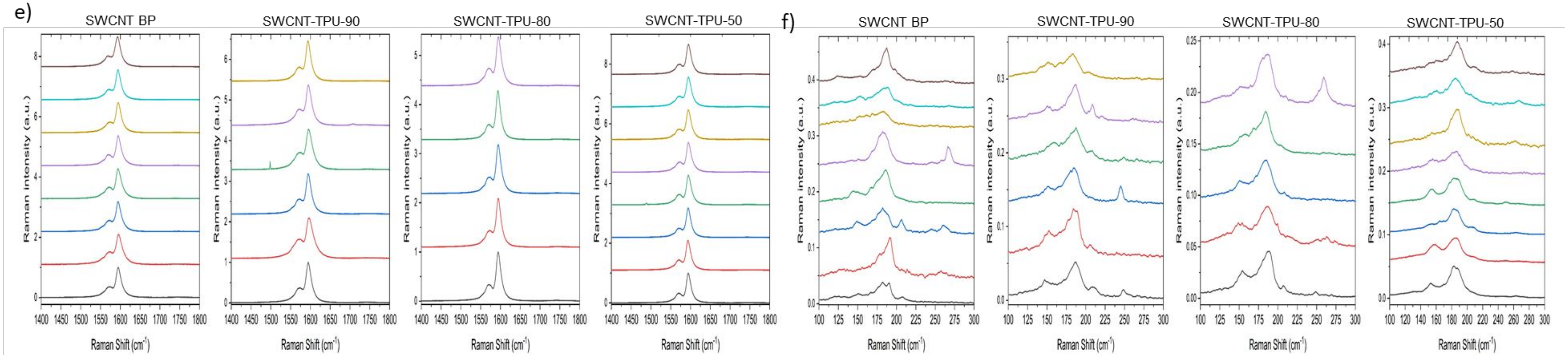

Figure S4. Characterization of SWCNT buckypaper (BP) and SWCNT-TPU nanocomposite sheets by Raman spectroscopy: Comparison of representative Raman spectra with typical RBM, G, D, and G' bands for SWCNT (a) with zoomed region showing D & G band region (b) and RBM region (c). (d) shows summary of G-band maximum position and I_D/I_G. (e) and (f) show the G and RBM regions, respectively, for all individual spectra measured at multiple spots on each sample.

Table S2. Recent advances in thermoelectric properties of carbon nanotube/TPU based composites based on literature reports

Sample	Filler	Content	Processing	σ	S	PF	ZT	E	σ_{fail}	ε_{fail}	Ref.
		(wt%)		(S cm^{-1})	(μV K^{-1})	(μW·m^{-1}·K^{-2})	(-)	(MPa)	(MPa)	(%)	
MWCNT/PEDOT:PSS/PU	MWCNT CoMoCAT®	20	Solution Processing	1.4	10	1.4	–	–	–	–	[2]
PPBH[I]/SWCNT/PUBI[II]	SWCNT Tuball™	10	Drop casting	110	24	5.2 × 10^{-1}	–	–	–	3.8	[3]
PEDOT:PSS/CNT/WPU[III]	SWCNT Chengdu Organic Chemicals	50	Drop casting	19	31	–	–	–	–	400	[4]
MWCNT/TPU	MWCNT Nanocyl® NC7000	5	Melt Mixing	0.45	8.5	4 × 10^{-3}	–	25	9.5	154	[5]
MWCNT/TPU	L-MWCNT Nanocyl®	5	Melt Mixing	0.94	18.5	4 × 10^{-2}	1.42 × 10^{-5}	28.5	10.4	161	[5]
MWCNT/PVA[IV]/PU	MWCNT Chengdu Organic Chemicals		Wet Spinning	4	17	1.16 × 10^{-1}	–	–	–	–	[6]
SWCNT/PVA/PU	SWCNT Chengdu Organic Chemicals		Wet Spinning	10	19	3.6 × 10^{-1}	–	–	–	–	[6]
SWCNT/PVA/PU	SWCNT Nanjing XFNANO Materials		Wet Spinning	9	38	1.3	–	–	–	–	[6]
PEDOT:PSS/CNT/PCL[V]/ PU	SWCNT Chengdu Organic Chemicals	7:3 (PEDOT:PSS) :(SWCNT)	Electro-spinning and filtration	16	35	1.9	–	–	–	400	[7]
Graphene/CNF[VI]/TPU			Direct Ink Writing	–	30.8	–	–	–	–	–	[8]
SWCNT/PVP[VII]/PU	SWCNT Chengdu Organic Chemicals	7:3 (SWCNT): (PVP)	Electrospin-ning/Spray Technology	20	51	5	–	–	–	250	[9]
SWCNT/TPU	***SWCNT Tuball™***	***50 (15 vol%)***<br><br>***90 (16 vol%)***	***Solution Mixing/one step filtration***	***133***<br><br>***157***	***65.5***<br><br>***64.5***	***56.9***<br><br>***65.4***	***2.4 × 10^{-3}***<br><br>***2.9 × 10^{-3}***	***1773***<br><br>***576***	***80***<br><br>***14***	***41***<br><br>***11***	***This work***

[I] Polymer particles bearing many small bumps and crosslinkable hydroxyl groups on their surfaces (PPBH). [II] A waterdispersible polyurethane with blocked terminal isocyanate groups (PUBI).

III Waterborne polyurethane. IV Poly(vinyl alcohol). V Polycaprolactone. VI Carbon nanofiber. VII Polyvinyl pyrrolidone

References:

1. Bârsan, O.A.; Hoffmann, G.G.; van der Ven, L.G.J.; de With, G. Single-Walled Carbon Nanotube Networks: The Influence of Individual Tube–Tube Contacts on the Large-Scale Conductivity of Polymer Composites. *Advanced Functional Materials* **2016**, *26*, 4377-4385, doi:https://doi.org/10.1002/adfm.201600435.
2. Wu, Q.; Hu, J. Waterborne polyurethane based thermoelectric composites and their application potential in wearable thermoelectric textiles. *Composites Part B: Engineering* **2016**, *107*, 59-66, doi:https://doi.org/10.1016/j.compositesb.2016.09.068.
3. Xiao, C.; Xue, Y.; Liu, M.; Xu, X.; Wu, X.; Wang, Z.; Xu, Y.; Chen, G. Polymer composites with lychee-like core covered by segregated conducting and flexible networks: unique morphology, high flexibility, stretchability and thermoelectric performance. *Composites Science and Technology* **2018**, *161*, 16-21, doi:https://doi.org/10.1016/j.compscitech.2018.03.039.
4. He, X.; Hao, Y.; He, M.; Qin, X.; Wang, L.; Yu, J. Stretchable Thermoelectric-Based Self-Powered Dual-Parameter Sensors with Decoupled Temperature and Strain Sensing. *ACS Applied Materials & Interfaces* **2021**, *13*, 60498-60507, doi:10.1021/acsami.1c20456.
5. Tzounis, L.; Petousis, M.; Grammatikos, S.; Vidakis, N. 3D Printed Thermoelectric Polyurethane/Multiwalled Carbon Nanotube Nanocomposites: A Novel Approach towards the Fabrication of Flexible and Stretchable Organic Thermoelectrics. *Materials* **2020**, *13*, 2879.
6. Zhang, C.; Zhang, Q.; Zhang, D.; Wang, M.; Bo, Y.; Fan, X.; Li, F.; Liang, J.; Huang, Y.; Ma, R.; et al. Highly Stretchable Carbon Nanotubes/Polymer Thermoelectric Fibers. *Nano Letters* **2021**, *21*, 1047-1055, doi:10.1021/acs.nanolett.0c04252.
7. He, X.; Shi, J.; Hao, Y.; Wang, L.; Qin, X.; Yu, J. PEDOT:PSS/CNT composites based ultra-stretchable thermoelectrics and their application as strain sensors. *Composites Communications* **2021**, *27*, 100822, doi:https://doi.org/10.1016/j.coco.2021.100822.
8. Yin, Y.; Wang, Y.; Li, H.; Xu, J.; Zhang, C.; Li, X.; Cao, J.; Feng, H.; Zhu, G. A flexible dual parameter sensor with hierarchical porous structure for fully decoupled pressure–temperature sensing. *Chemical Engineering Journal* **2022**, *430*, 133158, doi:https://doi.org/10.1016/j.cej.2021.133158.
9. He, X.; Shi, J.; Hao, Y.; He, M.; Cai, J.; Qin, X.; Wang, L.; Yu, J. Highly stretchable, durable, and breathable thermoelectric fabrics for human body energy harvesting and sensing. *Carbon Energy* **2022**, *4*, 621-632, doi:https://doi.org/10.1002/cey2.186.
